# Development of a Fluorescein-Based Probe with an “Off–On” Mechanism for Selective Detection of Copper (II) Ions and Its Application in Imaging of Living Cells

**DOI:** 10.3390/bios13030301

**Published:** 2023-02-21

**Authors:** Yinjuan Bai, Hongpeng Zhang, Bingqin Yang, Xin Leng

**Affiliations:** 1 College of Chemistry & Materials Science, Northwest University, Xi’an 710127, China; 2 College of Science, Northwest University, Xi’an 710069, China

**Keywords:** fluorescein-based probe, complexation mechanism, copper ion detection, bioimaging

## Abstract

Copper is a common metallic element that plays an extremely essential role in the physiological activities of living organisms. The slightest change in copper levels in the human body can trigger various diseases. Therefore, it is important to accurately and efficiently monitor copper ion levels in the human body. Recent studies have shown that fluorescent probes have obvious advantages in bioimaging and Cu^2+^ detection. Therefore, a novel Cu^2+^ probe (N2) was designed and synthesized from fluorescein, hydrazine hydrate and 5-p-nitrophenylfurfural that is sensitive to and can detect Cu^2+^ within 100 s. The response mechanism of the N2 probe to Cu^2+^ was studied by several methods such as Job’s plots and MS analysis, which showed that the Cu^2+^ and the N2 probe were coordinated in a complexation ratio of 1:1. In addition, compared with other cations investigated in this study, the N2 probe showed excellent selectivity and sensitivity to Cu^2+^, exhibiting distinct fluorescence absorption at 525 nm. Furthermore, in the equivalent range of 0.1–1.5, there is a good linear relationship between Cu^2+^ concentration and fluorescence intensity, and the detection limit is 0.10 μM. It is worth mentioning that the reversible reaction between the N2 probe and Cu^2+^, as well as the good biocompatibility shown by the probe in bioimaging, make it a promising candidate for Cu^2+^ biosensor applications.

## 1. Introduction

Copper is commonly found in various organisms and is one of the more abundant transition metals in the human body [1,2]. Compared with other transition metals, Cu has a strong stability constant of binding ligands, which makes it an ideal enzyme cofactor [3,4]. Cu can enter the human body in a variety of ways, such as through inhalation, diet and environmental intake [5]. According to some studies, Cu^2+^ solutions and metallic Cu can effectively inhibit the growth of viruses and even inactivate them [6,7]. However, for human body, systematic Cu content must be intermediate at a relatively fixed level [8]. Changes in Cu levels can contribute to anemia, coronary heart disease, Wilson’s disease and Alzheimer’s disease and can also trigger cell carcinogenesis and apoptosis [3,8,9,10]. Levels of Cu have been reported to be a sign of cancers, such as breast cancer, which can be measured by Cu^2+^ levels, and prostate tumor proliferation, which can be inhibited by reducing the uptake of Cu [11]. Therefore, accurate detection of the content of Cu^2+^ in organisms is required.

Currently, Cu^2+^ is detected by atomic absorption spectrometry [12], inductively coupled plasma mass spectrometry [13], electrochemical methods [14], etc. In these detection methods, it is indubitably necessary to involve advanced pretreatment processes and several large-scale instruments, which limit rapid detection in the wild and nondestructive functions of organisms [15,16,17,18]. Fluorescent probes have been widely used in the identification and measurement of Cu^2+^ due to their advantages of sensitivity, good biocompatibility, simple operation, and fast and specific recognition [19,20,21,22,23,24,25]. As a consequence, a series of fluorescent probes based on traditional fluorophores, such as coumarin, rhodamine and BODIPY, has been explored extensively [26,27,28,29,30,31,32]. Luciferin, a kind of optically stable fluorophore, has also been the subject of broad research [33,34,35]. It has been shown that amide-modified fluorescein derivatives with large metal ion coordination sites can be complexed with metal ions, resulting in changes in the fluorescence of the probe and successful applications in bioimaging [20,36,37,38].

**Figure 1:**
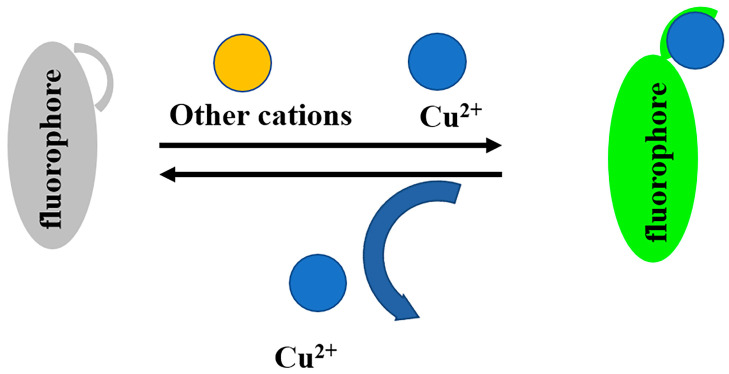
The “off–on” mechanism of the probe in response to Cu^2+^.

In this study, we designed and synthesized a novel fluorescein derivative of Cu^2+^ fluorescence, the N2 probe. It is worth noting that this probe has a unique ability to recognize Cu^2+^ in multi-ion coexistence solutions and shows a very intense fluorescence absorption peak at 525 nm, unlike other ions that are almost unchanged. Compared with other probes reported in the published literature [25,39,40,41,42,43], this probe has a relatively low detection limit and a stabilized fluorescence response at pH 6.0–9.0. In addition, through the discussion of the response mechanism between Cu^2+^ and the probe, it was revealed that Cu^2+^ enabled a certain reversibility of the recognition effect of the N2 probe by opening the lactam ring of the N2 probe and complexing with it in a 1:1 coordination ratio (Figure 1). Furthermore, cytotoxicity testing showed that the N2 probe has good biocompatibility. Bioimaging showed that N2 probe can be used as an intracellular Cu^2+^ tracer sensing material.

## 2. Materials and Methods

### 2.1. Chemical Reagents

Hydrochloric acid, copper sulfate, sodium hydrate, hydrazine hydrate, fluorescein, ethanol and 5-p-nitrophenyl furfural were purchased from Aladdin Reagent Co., Ltd. (Shanghai, China). All such chemical reagents were used with no additional purification.

### 2.2. Apparatus and Instrumentation

ZF-C-type UV-vis spectrophotometer, a Bruker Tensor 27 spectrometer, a Hitachi F-4500 fluorescence spectrophotometer, a Bruker micro TOF-Q II ESI-TOF LC/MS/MS spectrometer, a Varian INOVA-400 MHz spectrometer (400 MHz), a Spectra max190 molecular devices and an Olympus FV1000 confocal microscope were used in this research.

### 2.3. The Synthesis of N2

According to the literature [44], the synthesis process scheme was exhibited in Scheme S1. Fluorescein hydrazine was synthesized from fluorescein and hydrazine hydrate. First 6.00 g (18.05 mmol) of fluorescein was added to a 250 mL three-necked round-bottom flask and dissolve it by adding 110 mL of anhydrous ethanol. Then, 8.0 mL of hydrazine hydrate was slowly added to the solution over the course of about 30 min. The temperature of the reaction was gradually raised to 80 °C, and the reaction solution was refluxed for 12 h. After the reaction was finished, the solution was cooled to room temperature, and the reaction solvent was evaporated under reduced pressure. Then, 500 mL of water was added, and the pH was adjusted to 4–5 with concentrated hydrochloric acid. The pH of the system was continuously adjusted to 9–10 with sodium hydroxide. The solid was filtered under reduced pressure, washed 3 times with distilled water and dried to obtain 5.88 g of light-yellow solid.

Then, 80 mL of fluorescein hydrazine (3.59 g, 10.36 mmol) dissolved in anhydrous ethanol and 50 mL of 5-p-nitrophenyl furfural (1.50 g, 6.90 mmol) dissolved in anhydrous ethanol were added to a 250 mL round-bottom flask. The solution was heated to 78 °C for reaction, and TLC, ethyl acetate and petroleum ether (*v*/*v* = 5/3) were used to monitor the reaction process. The reaction solution was refluxed for 3 h, and a large amount of solid was precipitated from the bottom of the bottle. After filtration under reduced pressure, a solid was obtained, which was washed with mother liquor 3 times and recrystallized with anhydrous ethanol. The crystallized solid was dried, and 3.65 g of an orange solid was obtained with a yield of 96.88% and a melting point of 243–245 °C.

^1^H NMR (400 MHz, TMS, DMSO-d_6_) *δ* 9.95 (s, 2H, OH), 8.77 (s, 1H), 8.32 (d, *J* = 9.0 Hz, 2H, NO_2_-Ph-H), 7.92 (d, *J* = 8.9 Hz, 2H, NO_2_-Ph-H), 7.93 (d, *J* = 6.4 Hz, 1H, Ph-H), 7.68–7.55 (m, 2H, Ph-H), 7.38 (d, *J* = 3.6 Hz, 1H, furan), 7.12 (d, *J* = 7.4 Hz, 1H, Ph-H), 6.96 (d, *J* = 3.7 Hz, 1H, furan), 6.70 (d, *J* = 2.3 Hz, 2H, OH-Ph-H), 6.54 (d, *J* = 8.6 Hz, 2H, OH-Ph-H), 6.47 (dd, *J*_1_ = 8.6 Hz, *J*_2_ = 2.3 Hz, 2H, OH-Ph-H). ^13^C NMR (100 MHz, TMS, DMSO-d_6_) *δ* 164.30, 159.17, 152.88, 152.64, 151.22, 151.08, 146.85, 137.69, 135.54,134.68, 129.63, 128.82, 128.28, 125.00, 124.16, 123.77, 116.89, 112.87, 110.34, 103.11, 65.85. HRMS(ESI) *m*/*z* calcd for C_31_H_19_N_3_O_7_Na(M+Na)^+^: 568.1115. Found: 568.1088. IR (KBr, cm^−1^): 3199.59(OH), 1690.90(C=O), 1629.08(C=N), 1610.82, 1505.02, 1461.85, 1362.82, 1329.06, 1262.24, 1214.70, 1185.62, 1105.48, 992.98, 970.98, 913.74, 848.16, 808.40, 793.08, 752.53, 689.79 (Figures S2–S4 in the Supplementary Material).

### 2.4. Colorimetric Determination of Copper Ions

To facilitate the titration experiments, the N2 probe, deionized water and EtOH were prepared as a 1 mM master mix. In the titration experiment, a set concentration gradient of Cu^2+^ was added to a 10 mL colorimetric tube containing 1.0 mL of 200 μM N2 probe master mix. Then, it was fixed to 10 mL with PBS solution. For the interference assay, 20 μM Cu^2+^ and 1.0 mL of 200 μM N2 probe master mix were mixed with 1.0 mL of the test substance, which was two equivalents of the probe N2, and the volume was fixated with PBS to 10 mL in a colorimetric tube. In the ethylenediamine titration context, 1.0 mL of 200 µM Cu^2+^, 1.0 mL of 200 µM N2 probe master mix and different amounts of ethylenediamine were added to a 10 mL colorimetric tube with PBS. Spectroscopic analysis was performed using a 1 cm cuvette. In various tests, absorbance at 440 nm and fluorescence intensity at 525 nm were recorded separately.

### 2.5. Detection Limit of the Probe

The detection limits were calculated based on the measured fluorescence signals. In this study, the luminescence intensity of N2 (20.0 µM) was multiplied by 10 to determine the ratio of δ/S and the standard deviation of the blank assay. Under this condition, there was a good linear relationship between the relative luminescence intensity (525 nm) and the concentration of Cu^2+^ in the range of 10.0–40.0 µM. The detection limit was determined by the following equation: detection limit = K × δ/S, where S is the gradient of the concentration and the intensity of the sample, and δ is the standard deviation of the blank determination. Fluorescence analysis showed: y = 124.41x + 111.28 (R^2^ = 0.983), *δ* = 4.147 (N = 10), S = 124.41, K = 3; LOD = 3 × 4.147/124.41 = 0.10 µM.

### 2.6. Cytotoxicity Study

The CCK-8 method was carried out to analyze cytotoxicity. Different concentrations of probes (0 μM, 2.5 μM, 5 μM, 10 μM, 20 μM and 40 μM) were added into the cells, which had been cultured at 37 °C in 96-well plates for 24 h. The absorbance of the cells was measured at 450 nm, combined with CCK-8 and incubated for two hours. The above experiments were repeated three times, and the results of cytotoxicity were presented as a percentage of control cells.

### 2.7. Cell Culture Experiment and Cell Imaging

MCF-7 cells were digested in trypsin containing 0.25% EDTA. When a tendency of rounding and floating was observed under the microscope, DMEM complete medium (89% DMEM medium, 10% FBS, 1% penicillin-streptomycin) was added to terminate the digestion, and the supernatant was centrifuged and separated on a centrifuge. Then, DMEM complete medium was added to afford a given concentration of cell suspension, divided equally into three confocal dishes and incubated in a 5% CO_2_ incubator at 37 °C for 24 h. Then, 1 mL of PBS buffer was added, and confocal microscopic imaging was performed. Then, the configured probe solution was added and incubated in the incubator for 20 min, at which time the concentration of the fluorescent probe in the confocal Petri dishes was about 40 μmol/L. After washing three times with PBS buffer, imaging was performed under confocal microscopy in a wavelength channel of 488 nm. The cells were then incubated in a copper ion solution at a concentration of approximately 40 μmol/L for 20 min, washed three times with PBS and imaged. Fluorescence field and bright-field images were acquired separately and superimposed.

## 3. Results and Discussion

### 3.1. Effect of pH and Response Time

**Figure 2:**
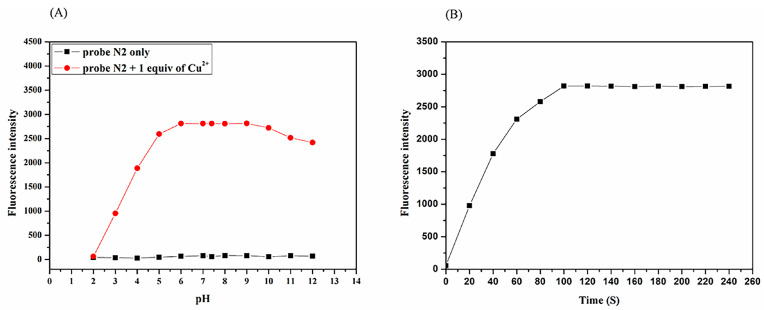
(**A**) Fluorescence intensity (525 nm) of the N2 probe (20.0 µM) and the combination of the probe and Cu^2+^ at different PH values. (**B**) Fluorescence intensity (525 nm) at different times after the addition of Cu^2+^ (20.0 μM) to the N2 probe (20.0 µM). λ_ex_ = 440 nm.

The influence of pH on N2 and N2 towards Cu^2+^ was evaluated in PBS buffer (10 Mm, PH = 7.4)/EtOH (1:1, *v*/*v*) (Figure 2A). It is apparent that the N2 probe has a relatively stable fluorescence response to Cu^2+^ in the pH range of 6.0 to 9.0. Hence, it can be adapted for bioimaging experiments at pH 7.4. The response times were examined at 525 nm (Figure 2B). After the addition of Cu^2+^ (20.0 μM), the fluorescence intensity at 525 nm intensified and reached a smoothed level after 100 s, which means that Cu^2+^ could be promptly detected by the N2 probe.

### 3.2. Probe Selection and Competition

**Figure 3:**
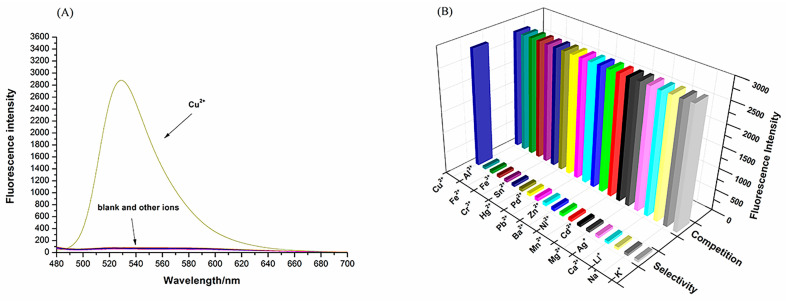
(**A**) Fluorescence spectrum of N2 (20 µM) in the presence of various metal ions, i.e., K^+^, Na^+^, Li^+^, Ca^2+^, Ag^+^, Mg^2+^, Cd^2+^, Mn^2+^, Ni^2+^, Ba^2+^, Zn^2+^, Pb^2+^, Pd^2+^, Hg^2+^, Sn^4+^, Cr^3+^, Fe^3+^, Fe^2+^, Al^3+^ and Cu^2+^ (20 µM), in PBS buffer (10 mM, pH = 7.4)/EtOH (1:1, *v*/*v*); λ_ex_ = 440 nm. (**B**) Fluorescence spectrum of N2 (20 µM) in the presence of various metal ions both alone and in combination, i.e., K^+^, Na^+^, Li^+^, Ca^2+^, Ag^+^, Mg^2+^, Cd^2+^, Mn^2+^, Ni^2+^, Ba^2+^, Zn^2+^, Pb^2+^, Pd^2+^, Hg^2+^, Sn^4+^, Cr^3+^, Fe^3+^, Fe^2+^, Al^3+^ and Cu^2+^ (20 µM), in PBS buffer (10 mM, pH = 7.4)/EtOH (1:1, *v*/*v*); λ_ex_ = 440 nm.

The sensing properties of the N2 probe towards various cations, such as K^+^, Na^+^, Li^+^, Ca^2+^, Ag^+^, Mg2^+^, Cd^2+^, Mn^2+^, Ni^2+^, Ba^2+^, Zn^2+^, Pb^2+^, Pd^2+^, Hg^2+^, Sn^4+^, Cr^3+^, Fe^3+^, Fe^2+^, Al^3+^ and Cu^2+^, in PBS buffer (10 mM, PH = 7.4)/EtOH (1:1, *v*/*v*) were examined to evaluate the selectivity and anti-interference ability of N2 to Cu^2+^. As shown in Figure 3, with the addition of Cu^2+^, a significant increase in fluorescence intensity was observed at 525 nm, in remarkably contrast to the other ions (Figure 3A). In the next competitive experiments, the fluorescence intensity of the probes changed slightly when other ions were added (Figure 3B). Thus, the N2 probe has good selectivity for this application.

### 3.3. Qualitative and Quantitative Studies

**Figure 4:**
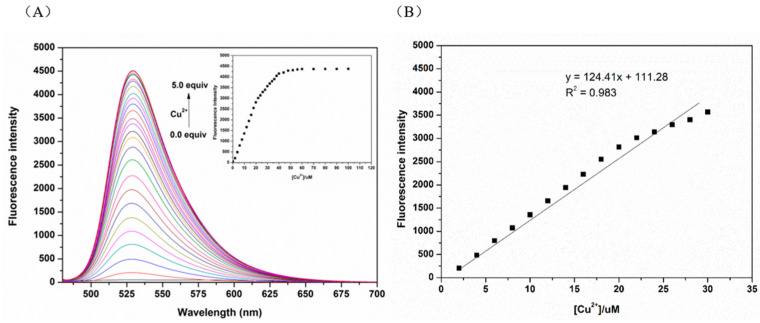
(**A**) Fluorescence titration of the N2 probe with different concentrations of Cu^2+^ (0.0 to 100.0 μM) (20.0 µM). (**B**) Linear relationship between Cu^2+^ concentration and fluorescence intensity at 525 nm; λ_ex_ = 440 nm.

Cu^2+^ with different molar ratios (0–100 μM) was added to the equimolar N2 probe (5 μM) solution configured with PBS buffer (10 mM, PH = 7.4)/EtOH (1:1, *v/v*). Figure 4A shows that the fluorescence intensity at 525 nm increased with increased Cu^2+^ concentration. When the Cu^2+^ concentration reached 2.4 eq, the fluorescence intensity reached the maximum and no longer increased. There is a good linear relationship between the fluorescence intensity of the N2 probe in response to Cu^2+^ and the concentration of Cu^2+^ in the range of 0.1–1.5 eq (Figure 4B). The LOD of the N2 probe for Cu^2+^ was calculated as 0.10μmol/L using the formula LOD = 3*σ*/K which implies that the N2 probe has good sensitivity for detection of Cu^2+^.

### 3.4. Proposed Sensing Mechanism

**Figure 5:**
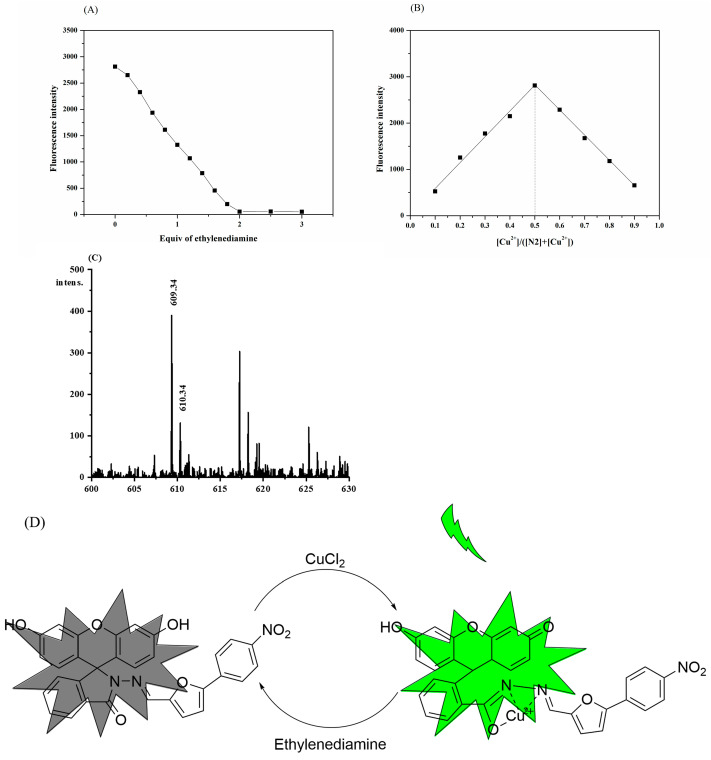
(**A**) The titration experiment of ethylenediamine and N2-Cu^2+^. (**B**) Job’s plot of the N2 probe and Cu^2+^. (**C**) MS Analysis of complex N2-Cu^2+^. (**D**) The proposed combination mode of N2 with Cu^2+^.

Methods such as Job’s plots and MS analysis were applied to further investigate the response mechanism of the N2 probe to Cu^2+^. The reversible complex reaction of the probe to Cu^2+^ was confirmed in an ethylenediamine titration experiment (Figure 5A), with the complexation ratio of 1:1 shown in the Job’s plots (Figure 5B).

In addition, the peak position of [C_31_H_19_CuO_7_N_3_ (M + H)]^+^ at *m*/*z* 610.34 in the mass spectrum can be matched with the signal of the N2 probe and Cu^2+^, which is consistent with the 1:1 coordination mechanism mentioned earlier (Figure 5C). This indicates that during the process of recognizing Cu^2+^, the amide bond broke, and a new Cu-O bond was formed. In summary, the complexation mechanism of the N2 probe in response to Cu^2+^ can be described by Figure 5D.

### 3.5. Cell Imaging

**Figure 6:**
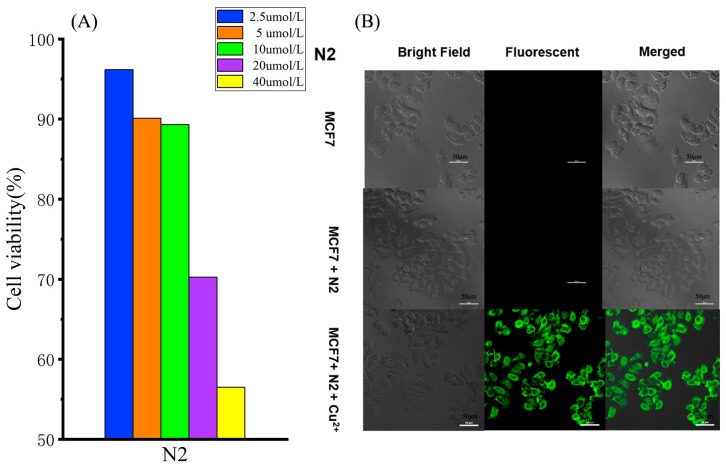
(**A**) A CCK-8 assay was performed on MCF-7 cells in the presence of different concentrations of N2 (2.5µM, 5 µM, 10 µM, 20 µM and 40 µM). (**B**) Bioimaging of MCF-7 cells after incubation with N2 (20.0 µM) in the absence and presence of Cu^2+^ (40.0 µM).

Based on the excellent properties of the probe, we investigated its bioimaging properties in cells. First, the cytotoxicity of the probe to MCF-7 cells was investigated by the CCK-8 method. MCF-7 cells were cultured in probe solutions of different concentrations (0–40 μM) for 24 h. As shown in Figure 6A and Table S1 in the Supplemetary Material, the probe had low cytotoxicity. Then, the bioimaging properties of the probe were examined. MCF-7 cells were cultured in the probe solution (40 μmol/L) for 20 min. No fluorescence was detected under confocal microscopy. After being treated with Cu^2+^ (40 μM) at 37 °C for 20 min, the cells showed obvious green fluorescence under the excitation of 448 nm light. Figure 6 also shows that the probe stained the cells but did not enter the nuclei. This further indicates that the N2 probe has good biocompatibility and a tracing effect on intracellular Cu^2+^.

## 4. Conclusions

This study describes a novel fluorescent probe (N2), the superior selectivity for Cu^2+^ of which makes it suitable for Cu^2+^ detection applications. Analysis of data from ethylenediamine titration experiments and mass spectrometry also revealed that the probe complexes with Cu^2+^ in a 1:1 coordination ratio. Moreover, the probe achieves a reversible fluorescence response to Cu^2+^ via a switching ring, which provides a potential idea for the design of reusable probes. Cell imaging shows that the probe has good biocompatibility and that the fluorescence response of the probe to Cu^2+^ is relatively stable at pH 6.0–9.0. In summary, the N2 probe can be used as a highly promising Cu^2+^ sensor in biological samples.

## Data Availability

The data presented in this study are available within the article.

## Supplementary Materials

The following supporting information can be downloaded at: https://www.mdpi.com/article/10.3390/bios13030301/s1, Scheme S1: Synthesis route of probe N2; Figure S1: 1H NMR spectrum of compound N2 in d6-DMSO; Figure S2: 13C NMR spectrum of compoundN2 in d6-DMSO; Figure S3: ESI-MS spectrum of N2; Figure S4: IR spectrum of N2; Table S1: Determination of the toxicity of different concentrations of probes on human mammary cells.
