# Supplementary material for: Development of a Fluorescein-Based Probe with an “Off–On” Mechanism for Selective Detection of Copper (II) Ions and Its Application in Imaging of Living Cells
Source: Biosensors (Basel). 2023 Feb 21;13(3):301. doi: 10.3390/bios13030301 (PMC10046790; doi:10.3390/bios13030301)
Supplement: Supplementary file 1 [file biosensors-13-00301-s001.zip › biosensors-2165712-supplementary.pdf]

biosensors

Development of a Fluorescein-based Probe with an "off–on" Mechanism for Selective Detection of Copper(II) ions and Its Application in Imaging of Living Cells

CONTENTS

1. **Synthesis of Probe N2**
2. **LOD calculation**
3. **CCK experiment**

1. Synthesis of the N2 Probe

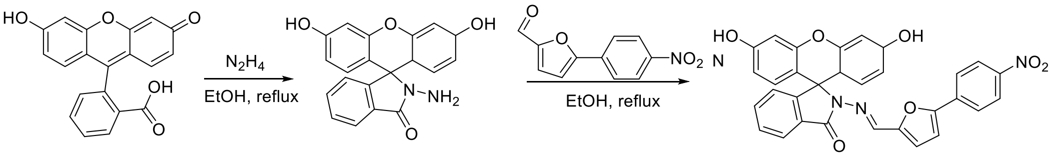

Scheme S1. Synthesis route of the N2 probe.

According to the literature[1], fluorescein hydrazine was synthesized from fluorescein and hydrazine hydrate. To this end, 6.00 g (18.05mmol) of fluorescein dissolved by 110 mL of anhydrous ethanol to a 250 mL three-neck flask; then, 8.0 mL of hydrazine hydrate was added slowly for about 30 minutes. The temperature was raised to 80 °C, and the reaction was refluxed for 12 h. After finishing, the solution was cooled to room temperature, and the solvent was evaporated. Then, 500 ml of water was added, and the pH was adjusted to 4-5 with hydrochloric acid. The pH was continually adjusted with sodium hydroxide to 9-10. The filter cake was washed three times and dried to obtain 5.88 g of a light-yellow solid.

Fluorescein hydrazide (3.59 g, 10.36 mmol) dissolved in 80 mL of anhydrous ethanol and 5-dissolved nitrofurfural (1.50 g, 6.90 mmol) dissolved in 50 mL of anhydrous ethanol were added to a 250 mL round-bottom flask. The reaction was heated to 78 degrees C, refluxed for 3 h and monitored by TLC. The reaction solution was frozen at the end of the reaction, and a large amount of solid was obtained from the bottom of the bottle. The filtrate was filtered under reduced pressure, washed three times with mother liquor and recrystallized with anhydrous ethanol. The solids were dried to afford 3.65 g of an orange solid with a yield of 96.88%.

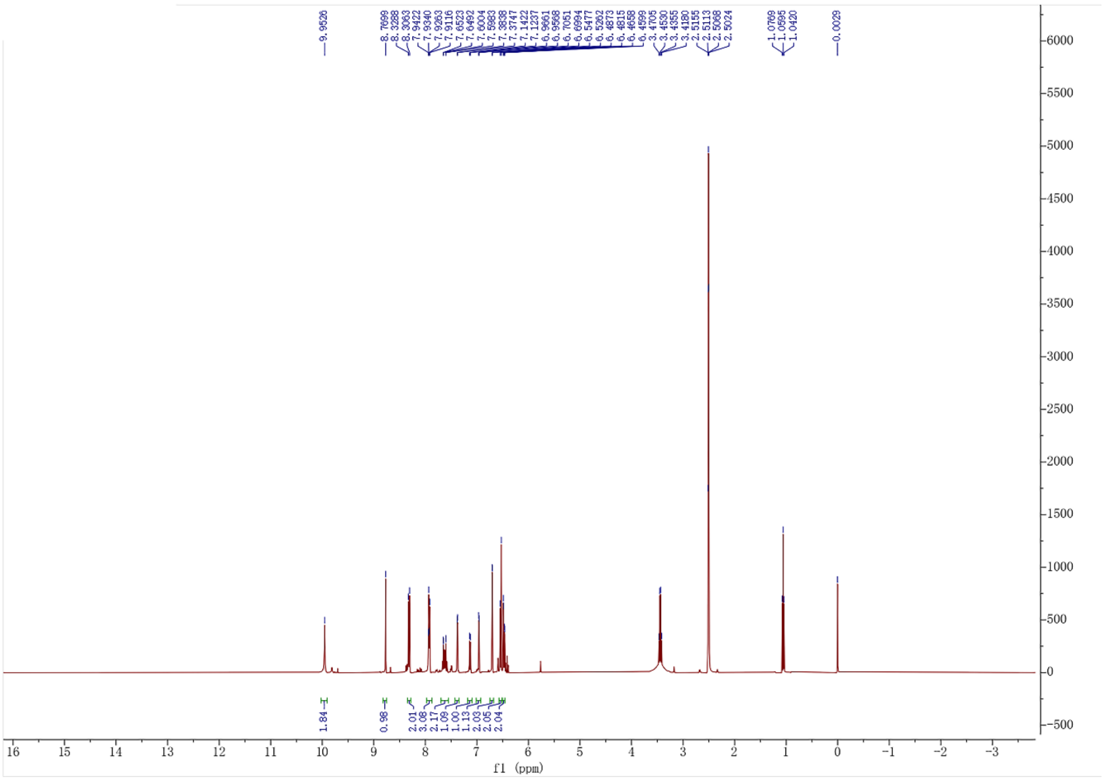

Figure S1. ^{1}H NMR spectrum of the N2 probe in d_6-DMSO.

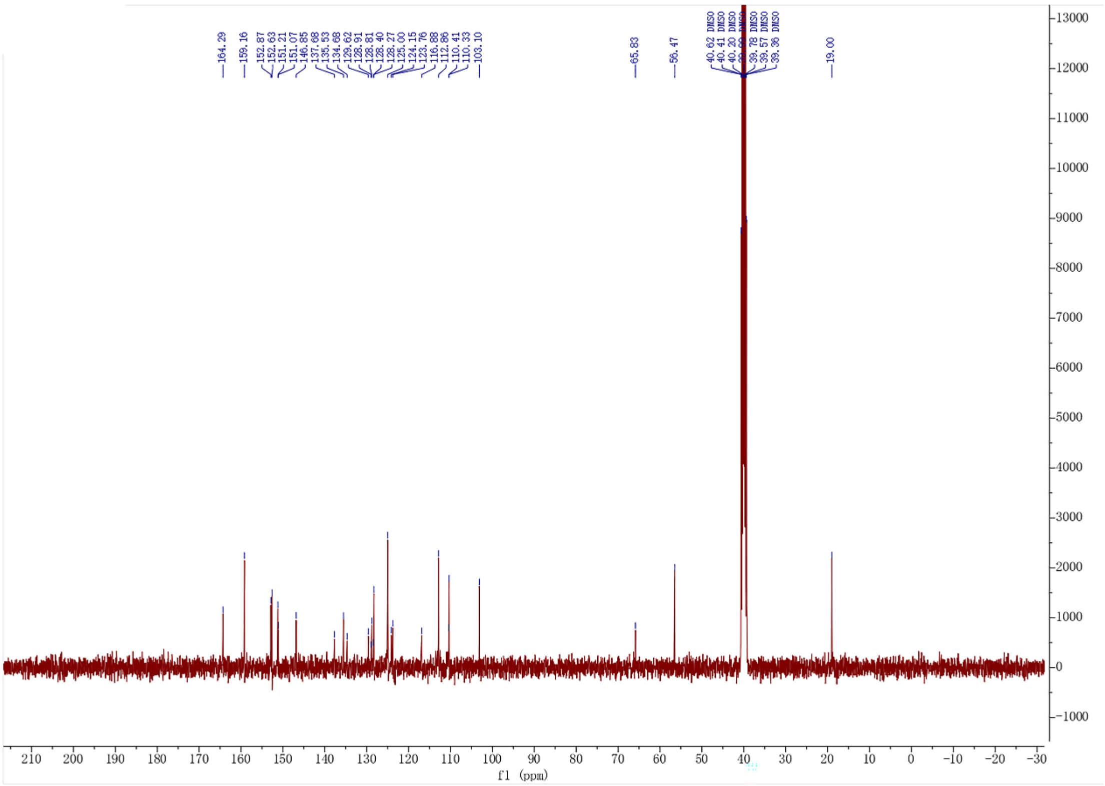

Figure S2. ^{13}CNMR spectrum of the N2 probe in d_6-DMSO.

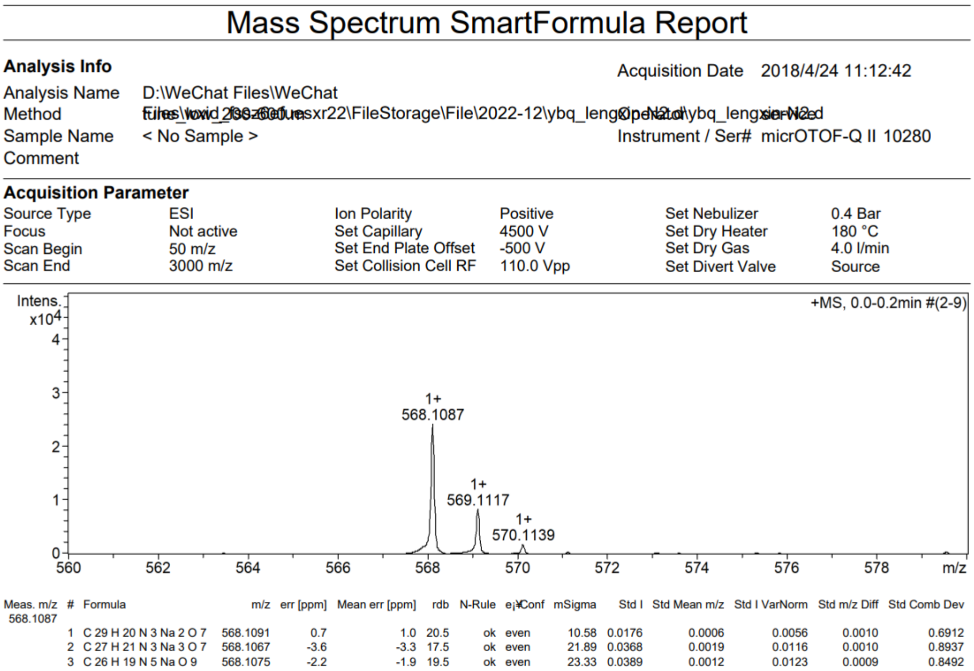

Figure S3. ESI-MS spectrum of N2.

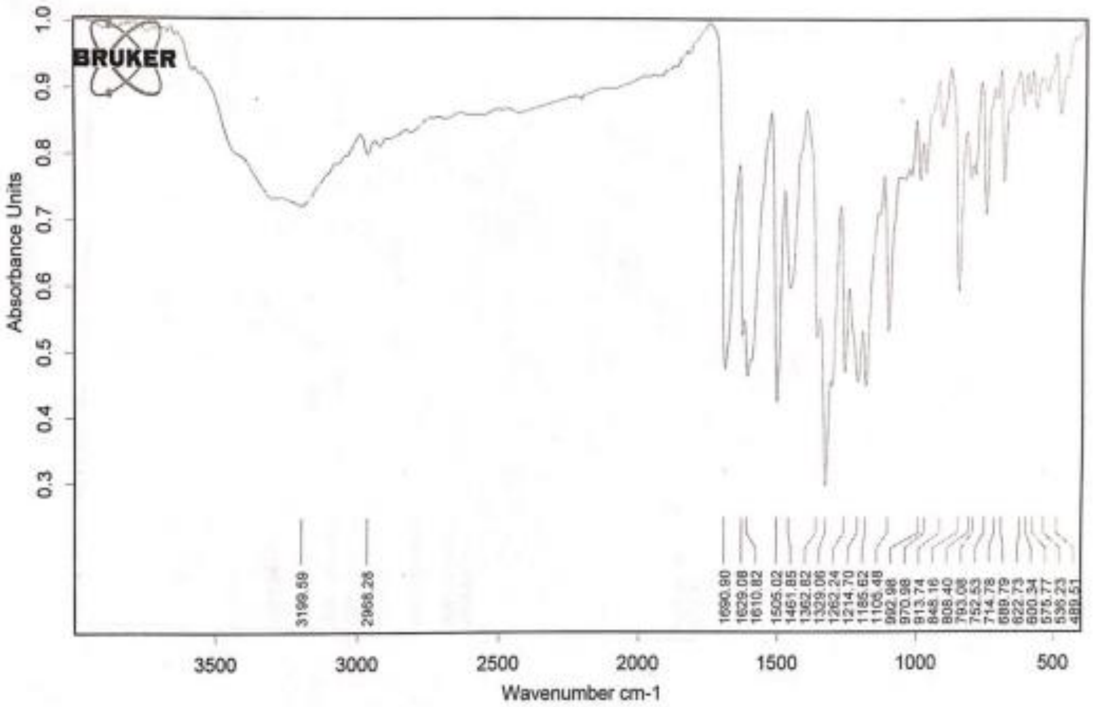

Figure S4. IR spectrum of N2.

2. LOD calculation

The detection limit was calculated according to the following equation: detection limit = K × δ/S, where S is the gradient of the concentration and the intensity of the sample, δ is the standard deviation of the blank determination. Fluorescence analysis showed: y =

124.41x+111.28（R^2 = 0.983）, δ= 4.147 (N= 10), S= 124.41, K= 3; LOD= 3x4.147/124.41 =0.10 μM.

3. CCK experiment

A (E)-3',6'-Dihydroxy-2(((5-(4-nitrophenyl)furan-2-yl)methylene)amino)spiro[isoindoline-1,9'-xanthen]-3-one (CCK) assay was conducted. MCF-7 cells were seeded in 96-well plates at a density of 5,000-10,000 cells/well and incubated in a 5% CO2/95% air incubator at 37 °C for 24 h. Then, 100 μL of different concentrations of N2 medium was added and incubated for 24 h. Next, 100 μL of CCK solution (CCK-8:CMEM v: v 1:9) was added to each well. After incubation in the culture chamber for 2 h, the absorbance of each well was recorded at 450 nm using an Elx800 absorbance microplate reader.

Table S1. Determination of the toxicity of different concentrations of probes on human mammary cells.

**Sample**	**2.5 μmol/L**	**5 μmol/L**	**10 μmol/L**	**20 μmol/L**	**40 μmol/L**
N2	96.2	90.189.3	89.3	70.2	56.5

Reference

1. Leng; Wang; Mi; Zhang; Yang; Chen, Novel Fluorescence Probe toward Cu(2+) Based on Fluorescein Derivatives and Its Bioimaging in Cells. *Biosensors (Basel)* **2022,** *12*.
